# Mesenchymal stem cells inhibited the differentiation of MDSCs via COX2/PGE2 in experimental sialadenitis

**DOI:** 10.1186/s13287-020-01837-x

**Published:** 2020-07-29

**Authors:** Jingjing Qi, Xiaojun Tang, Wenchao Li, Weiwei Chen, Genhong Yao, Lingyun Sun

**Affiliations:** 1 Department of Rheumatology and Immunology, The Affiliated Drum Tower Hospital of Nanjing University Medical School, Nanjing, 210093 People’s Republic of China; 2 Department of Immunology, College of Basic Medical Science, Dalian Medical University, Dalian, 116044 People’s Republic of China

**Keywords:** Mesenchymal stem cells, Myeloid-derived suppressor cells, Experimental sialadenitis, Prostaglandin E2

## Abstract

**Background:**

Mesenchymal stem cells (MSCs) can regulate innate and adaptive immune systems through interacting with immune cells directly and secreting multiple soluble factors. Due to their immunosuppressive properties, MSC transplantation has been applied to treat many clinical and experimental autoimmune diseases. However, the therapeutic effects and mechanisms by which MSCs regulate myeloid cells in Sjögren’s syndrome (SS) still remain elusive.

**Methods:**

The number and immune-suppressive activity of myeloid-derived suppressor cells (MDSCs), polymorphonuclear MDSCs (PMN-MDSCs), and monocytic MDSCs (M-MDSCs) were determined in non-obese diabetic (NOD) mice with sialadenitis and in NOD mice with human umbilical cord-derived MSC (UC-MSC) transplantation. Bone marrow cells were cultured with MSC-conditioned medium (MSC-CM) for 4 days. The number and immune-suppressive gene of MDSCs were detected by flow cytometry or qRT-PCR.

**Results:**

The results showed that the number of MDSCs and PMN-MDSCs was higher and M-MDSCs were lower in NOD mice with sialadenitis. UC-MSCs ameliorated SS-like syndrome by reducing MDSCs, PMN-MDSCs, and M-MDSCs and promoting the suppressive ability of MDSCs significantly in NOD mice. UC-MSCs inhibited the differentiation of MDSCs. In addition, UC-MSCs enhanced the suppressive ability of MDSCs in vitro. Mechanistically, MSCs inhibited the differentiation of MDSCs and PMN-MDSCs via secreting prostaglandin E2 (PGE2) and inhibited the differentiation of M-MDSCs through secreting interferon-β (IFN-β).

**Conclusions:**

Our findings suggested that MSCs alleviated SS-like symptoms by suppressing the aberrant accumulation and improving the suppressive function of MDSCs in NOD mice with sialadenitis.

## Background

Sjögren’s syndrome (SS) as a chronic systemic autoimmune disease is characterized by mononuclear infiltration in the exocrine glands, which results in salivary and lacrimal gland functional impairment [1]. Immunocomplex deposition caused by B cell hyperactivity implies the underlying pathogenesis [2], and other immune cell abnormal activations also display an important pathogenic role in SS pathological processes [3, 4]. Due to the complex syndrome of SS, clarifying the pathogenic mechanisms and improving the therapeutic strategy still are major challenges.

Mesenchymal stem cells (MSCs) are multipotent stem cells characterized by adhesive ability, fibroblast-like morphology, forming cell colonies, and differentiating into certain mesenchymal lineages [5–7]. MSCs have been described to reside in bone marrow, umbilical cord, adipose tissue, and a variety of other tissues [5] and have great potential in cell therapy for many pathologic conditions [8, 9]. MSCs can regulate the innate and adaptive immune systems by means of interacting with immune cells directly and secreting multiple soluble factors [10]. Human umbilical cord-derived MSCs (hUC-MSCs) as a reliable cellular source of MSCs have been applied to treat many experimental and clinical autoimmune diseases [11, 12]. In previous studies, hUC-MSCs ameliorated B6.*lpr* mice’s lupus symptoms by activating iNOS to inhibit Tfh cell expansion [13], hUC-MSCs ameliorated lupus nephritis by reducing macrophage infiltration and polarizing macrophage into an anti-inflammatory phenotype to prevent podocyte injury [14] and hUC-MSCs inhibit the differentiation of circulating Tfh cells via secreting indoleamine 2,3-dioxygenase (IDO) in SS patients [15]. However, the therapeutic effects and mechanisms of MSCs in SS need to be investigated further.

Myeloid-derived suppressor cells (MDSCs) are a heterogeneous population of immature myeloid cells generated under pathological states [16]. MDSCs are characterized by potent innate and adaptive immunity-suppressive activities by secreting inhibitory and anti-inflammatory cytokines, reactive oxygen (ROS), expressing arginase 1 (ARG-1), and inducible nitric oxide synthase (iNOS) [17, 18]. Two major different subpopulations of MDSCs (CD11b^+^Gr-1^+^) are monocytic MDSCs (M-MDSCs, CD11b^+^Ly6G^−^Ly6C^hi^) and polymorphonuclear MDSCs (PMN-MDSCs, CD11b^+^Ly6G^+^Ly6C^low^) in mice which are classified based on their phenotypic and morphological features [17, 19]. MDSCs were described to suppress antitumor immunity and facilitate tumor immune escape in patients with cancer [18]. However, accumulating evidences have revealed the non-immunological aspects of MDSCs. Studies showed that accumulated MDSCs played a critical pathogenic role in autoimmune arthritis [20] and MRL/*lpr* lupus mice [21] through decreasing Treg and driving Th17 cell differentiation. And increased and dysfunctional MDSCs promoted the development of Sjögren’s syndrome in salivary gland protein-immunized mice [22]. Moreover, our previous study found that excessive MDSCs exacerbated experimental Sjögren’s syndrome by inhibiting Th2 cell response [23]. However, the mechanisms of MSCs regulating myeloid cells in SS need to be explored.

Thus, in the present study, we detected the number and function of MDSCs, PMN-MDSCs, and M-MDSCs in non-obese diabetic (NOD) mice with or without SS-like symptoms and in hUC-MSC-treated or control NOD mice. In addition, we induced bone marrow (BM) cells from NOD mice to MDSCs with or without a MSC-conditioned medium. The results showed that hUC-MSCs ameliorated SS by inhibiting MDSC differentiation and improving their suppressive ability in NOD mice with sialadenitis.

## Methods

### Mice

Studies showed that non-obese diabetic (NOD) mice exhibited infiltration in the salivary and lacrimal glands and signature autoantibodies in serum [24]. Therefore, NOD mice were widely used to study experimental sialadenitis. Female NOD mice were obtained from the Model Animal Research Center of Nanjing University and kept under pathogen-free conditions in the animal center of the Affiliated Drum Tower Hospital of Nanjing University Medical School.

### Salivary flow rate

The salivary flow rate of mice was detected as described previously [23].

### Histological analysis

For histological analysis, submandibular gland (SG) tissue sections were stained with hematoxylin and eosin (H&E) after being fixed in 4% paraformaldehyde, embedded in paraffin, and sectioned at 3 μm. SG histological scores were determined based on the infiltration sizes and the degrees in the organization [25].

### Flow cytometry analysis

Bone marrow (BM) cells were flushed from mouse femurs and tibiae and prepared as single-cell suspension after lysing the red blood cells. Peripheral blood mononuclear cells (PBMC) in mouse blood samples were isolated with Ficoll-Hypaque (Axis-Shield). All cells labeled with antibodies were immediately analyzed on a FACS Calibur (BD Biosciences, Mountain View, CA, USA).

For the analysis of MDSCs, the appropriate number of cells was pre-incubated with optimal concentration surface marker antibodies (eBioscience), anti-mouse CD11b-APC, and anti-mouse Gr1-PE.

For the analysis of PMN-MDSCs and M-MDSCs, the appropriate number of cells was pre-incubated with optimal concentration surface marker antibodies (eBioscience), anti-mouse CD11b-APC, anti-mouse Ly6G-FITC, and anti-mouse Ly6C-PE.

For the analysis of immature or undifferentiated markers CD11c, F4/80, CD80, and MHCII on MDSCs, the appropriate number of cells was pre-incubated with optimal concentration surface marker antibodies (eBioscience), anti-mouse CD11b-APC, anti-mouse Gr1-PE or anti-mouse Gr1-FITC, and anti-mouse CD11c-FITC, anti-mouse F4/80-FITC, anti-mouse CD80-PE, or anti-mouse MHCII-PE.

### Expansion and adoptive transfer of hUC-MSCs

hUC-MSCs were prepared as described previously [26] and cultured at 37 °C in a 5% CO_2_ humidified atmosphere. Eight-week-old NOD mice were transferred with 1 × 10^6^ hUC-MSCs or equal volume PBS via the tail vein. All mice were sacrificed after 4 weeks.

### Preparation of hUC-MSC-conditioned medium

hUC-MSCs were cultured under a certain condition which has been used in mouse MDSC differentiation in vitro for 4 days. The culture supernatant of hUC-MSCs was collected and centrifuged at 300×*g* for 5 min at 4 °C to remove cellular debris. This supernatant was subsequently used as a MSC-conditioned medium (MSC-CM) [27].

### Bone marrow MDSC differentiation

Mouse BM cells were isolated and cultured as described previously [21] in RPMI-1640 with 10% fetal bovine serum (FBS, Gibco), 100 U/ml penicillin/streptomycin, 40 ng/ml IL-6, and 40 ng/ml GM-CSF (Pepro Tech) and with or without 50% MSC-CM or COX2 inhibitor NS-398 (10 μM) or anti-TGF-β1 (20 μg/ml) or anti-IFN-β (2.5 μg/ml) antibodies or PGE2 (10^−10^, 10^−8^, and 10^−6^ M) or IFN-β (10, 50, and 250 U/ml) (eBiosience) for 4 days, respectively.

### Quantitative RT-PCR

Gene expression analysis of mouse *gp91*^*phox*^, *arg-1*, *tgf-β1*, *inos*, and *Il-1β* and human *gro-α*, *gro-β*, *gro-γ*, *ido*, *cox2*, *tgf-β1*, *ifn-β*, and *Gapdh* was performed using SYBR-green-based quantitative RT-PCR (qRT-PCR). The expression level of each gene was calculated using relative standard curves and normalized to *Gapdh*. The primer sets were listed in the additional files (Table S1 and S2).

### Statistical analysis

All data are presented as means ± SEM and were analyzed with Student’s *t* test or one-way analysis of variance (ANOVA). *p* values less than 0.05 were considered statistically significant.

## Results

### hUC-MSCs decreased MDSCs in SS-like NOD mice

**Fig. 1:**
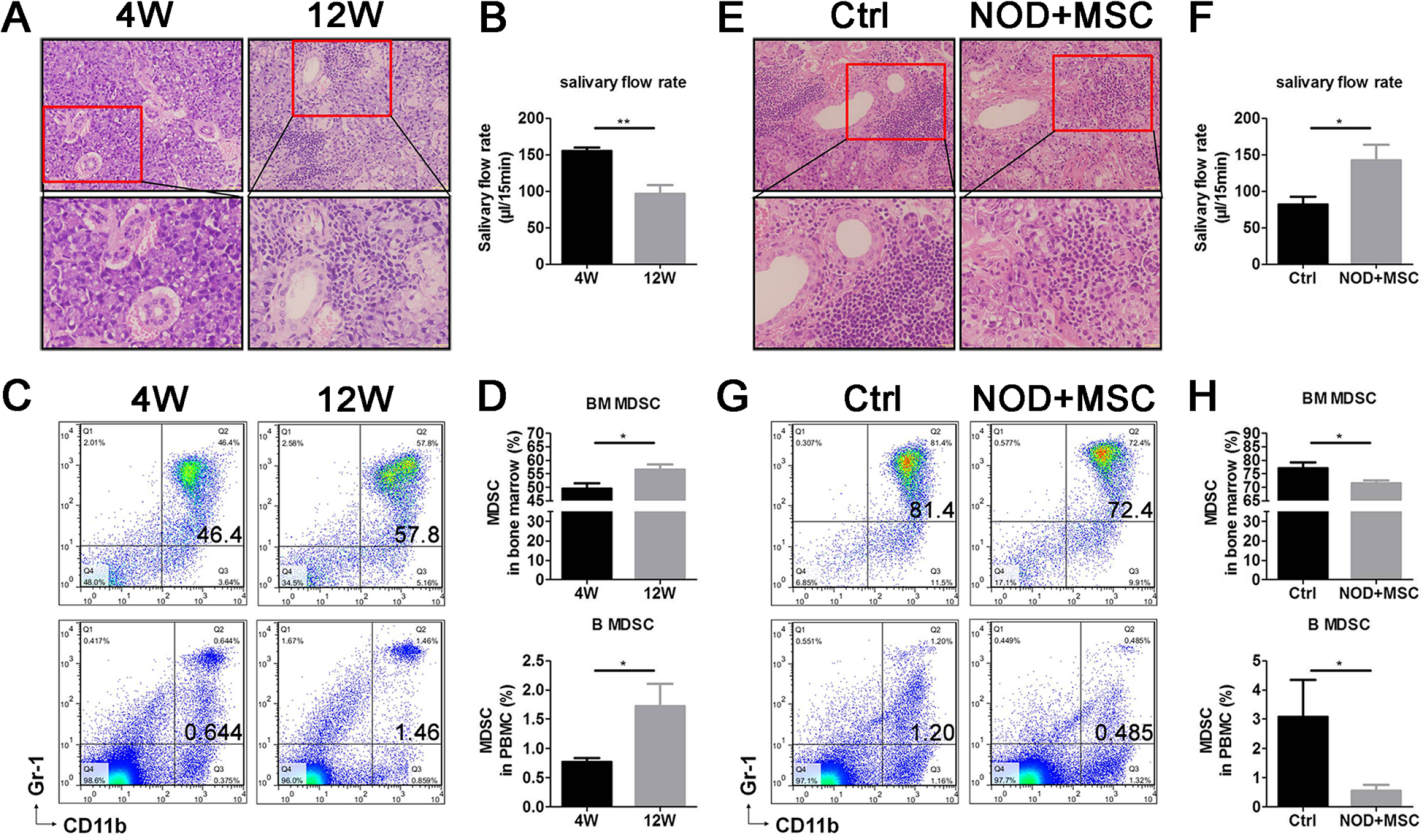
UC-MSCs ameliorated SS symptoms and decreased MDSCs in NOD mice. **a** Histological analysis of the submandibular glands (SG), **b** salivary flow rate, and **c** representative flow cytometry results and **d** percentage of BM and peripheral CD11b+Gr1+MDSCs in 4- and 12-week-old NOD mice. **e** Histological analysis of SG, **f** salivary flow rate, and **g** representative flow cytometry results and **h** percentage of BM and peripheral CD11b+Gr1+ MDSCs in UC-MSC transplantation and control NOD mice. Error bars indicate SEM. *p < 0.05, **p < 0.01, *n* = 5

In our previous study, MDSCs in the peripheral blood increased significantly during NOD SS-like pathological processes [23]. To determine the regulation of MSCs on MDSCs, here, we examined the SS-like symptoms and percentage of MDSCs in 4-week-old and 12-week-old NOD mice, as well as in hUC-MSC transplantation and control NOD mice. We found that 12-week-old NOD mice have severe infiltration in SG (Fig. 1a), lower salivary flow rate (Fig. 1b), and increased MDSCs in BM and PBMC (Fig. 1c, d) compared to 4-week-old NOD mice. After hUC-MSC transplantation, NOD mice showed fewer lymphocytic infiltration and less infiltration area in SG (Fig. 1e) and higher salivary flow rate (Fig. 1f). These results indicated that hUC-MSC transplantation improved the SS-like symptoms (the infiltration in SG was decreased, and the salivary flow rate was increased) in NOD mice. The results also showed that MDSCs in BM and PBMC decreased after MSC transplantation in NOD mice with sialadenitis (Fig. 1g, h). These results suggested that aberrant MDSC accumulation in BM and peripheral blood of NOD mice and hUC-MSCs ameliorated NOD SS-like symptoms effectively which may be in connection with decreased MDSCs in BM and peripheral blood.

### hUC-MSCs decreased PMN-MDSCs and M-MDSCs in NOD mice with sialadenitis

**Fig. 2:**
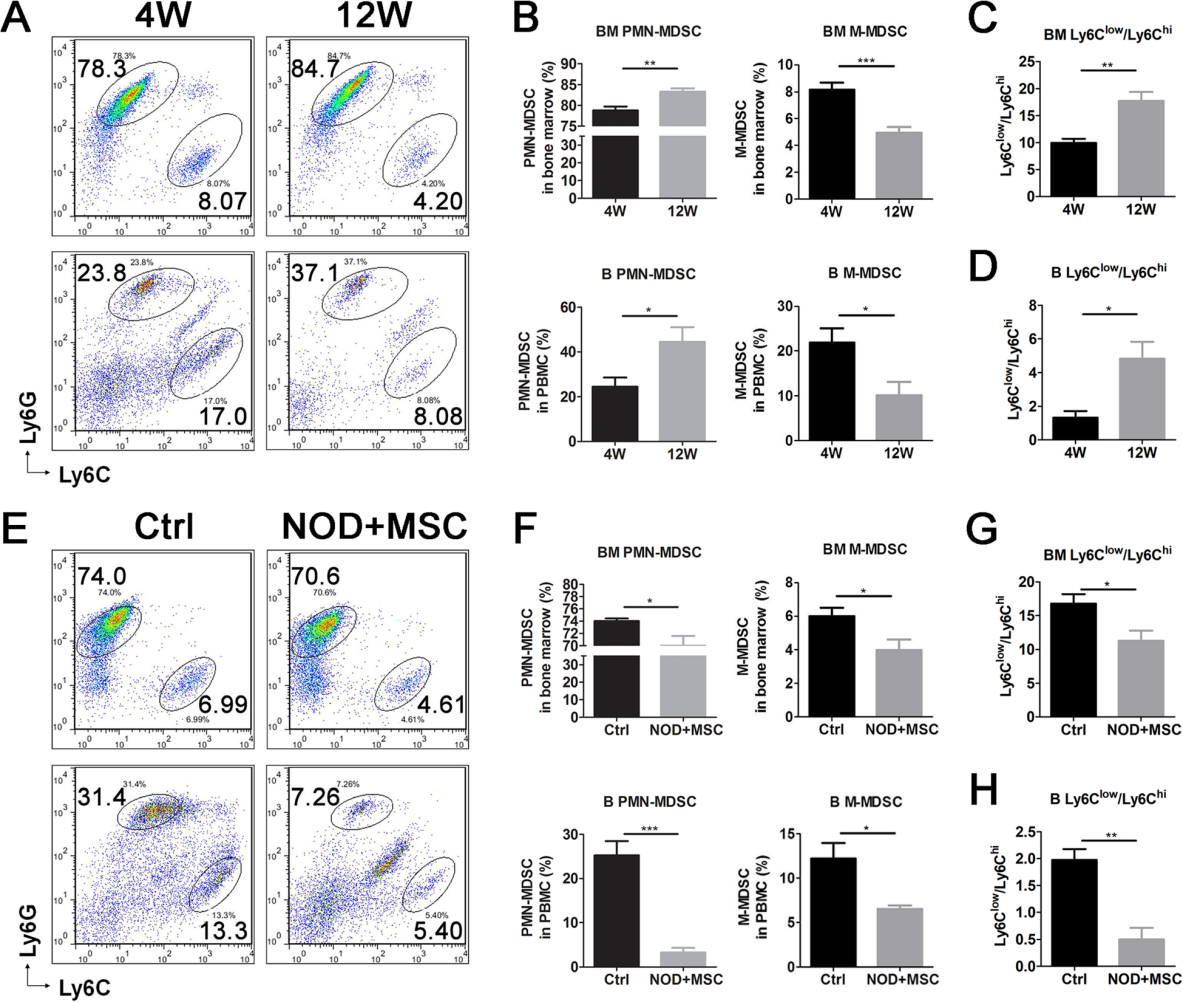
UC-MSCs decreased the frequency and ratio of PMN-MDSCs and M-MDSCs in SS-like NOD mice. **a**, **b** PMN-MDSCs (CD11b+Ly6G+Ly6Clow) and M-MDSCs (CD11b+Ly6G-Ly6Chi) were detected by flow cytometry in BM and PBMC from 4- and 12-week-old NOD mice. Ratio of PMN-MDSC (Ly6Clow)/M-MDSC (Ly6Chi) in **c** BM and **d** PBMC of 4- and 12-week-old NOD mice. **e**, **f** PMN-MDSCs and M-MDSCs were detected by flow cytometry in BM and PBMC from in UC-MSC transplantation and control NOD mice. Ratio of PMN-MDSC (Ly6Clow)/M-MDSC (Ly6Chi) in **g** BM and **h** PBMC of UC-MSC transplantation and control NOD mice. Error bars indicate SEM. *p < 0.05, **p < 0.01, ***p < 0.001, *n* = 5

Two major MDSC subsets PMN-MDSCs (CD11b^+^Ly6G^+^Ly6C^low^) and M-MDSCs (CD11b^+^Ly6G^−^Ly6C^hi^) may play different roles on suppressing T cell function in cancer and infectious and autoimmune diseases through different mechanisms [28]. Although 70–80% MDSCs are PMN-MDSCs, M-MDSCs more efficiently suppress T cell activation [29], whereas PMN-MDSCs did not display a measurable effect on T proliferation [30]. In this study, we detected the proportion of PMN-MDSCs and M-MDSCs in BM and PBMC of NOD mice. The results showed that 12-week-old NOD mice had more PMN-MDSCs, fewer M-MDSCs (Fig. 2a, b), and higher ratio of PMN-MDSCs/M-MDSCs (Fig. 2c, d) in BM and PBMC than 4-week-old NOD mice. The NOD mice with UC-MSC transplantation had lower PMN-MDSCs, M-MDSCs (Fig. 2e, f), and ratio of PMN-MDSCs/M-MDSCs (Fig. 2g, h) in BM and PBMC than control NOD mice. These results implied that both two MDSC subsets aberrantly accumulated and the ratio of PMN-MDSCs/M-MDSCs increased significantly in BM and PBMC of NOD mice. UC-MSCs decreased the percentage and ratio of PMN-MDSCs/M-MDSCs in BM and PBMC. These results indicated that hUC-MSCs restored the balance of PMN-MDSCs and M-MDSCs in NOD mice.

### hUC-MSCs improved suppressive activity of MDSCs in NOD mice

**Fig. 3:**
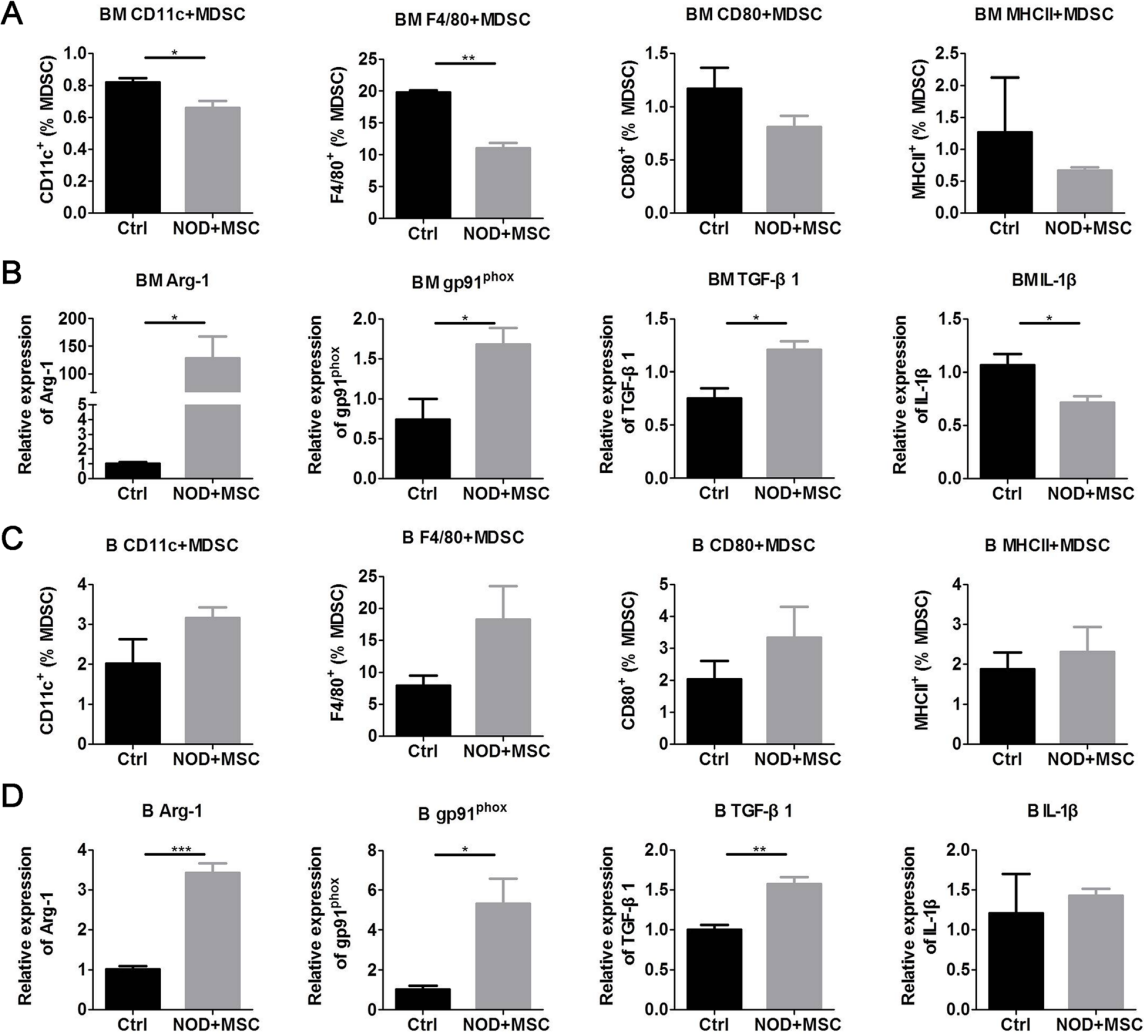
UC-MSCs improved the suppressive activity of MDSCs subsets in NOD mice. **a** Flow cytometry results of immature or undifferentiated markers CD11c, F4/80, CD80, and MHCII on BM MDSCs. **b** qPCR results of BM MDSC suppressive activity genes Arg-1, gp91phox, TGF-β1, and IL-1β. **c** Flow cytometry results of immature or undifferentiated markers CD11c, F4/80, CD80, and MHCII on PBMC MDSCs. **d** qPCR results of PBMC MDSC suppressive activity genes Arg-1, gp91phox, TGF-β1, and IL-1β. Error bars indicate SEM. *p < 0.05, **p < 0.01, *n* = 4

The expression of CD11c, F4/80, CD80, and MHCII on MDSCs reflects the immature or undifferentiated phenotype and the suppressive activity of antigen-specific immune responses [20, 28]. MDSCs can suppress the immune system via Arg-1, oxynitride, and TGF-β1 [17] and facilitate Th17 response by producing IL-1β [20, 21]. To evaluate the effects of hUC-MSCs on the suppressive ability of MDSCs, we detected the percentage of CD11c^+^, F4/80^+^, CD80^+^, and MHCII^+^MDSCs and the expression of Arg-1, gp91^phox^ (NOX components), TGF-β1, and IL-1β in MDSCs of BM and PBMC. We found that hUC-MSCs reduced CD11c^+^ and F4/80^+^MDSCs (Fig. 3a); increased Arg-1, gp91^phox^, and TGF-β1; and decreased IL-1β expression in BM (Fig. 3b). Although hUC-MSCs showed less effects on the CD11c, F4/80, CD80, and MHCII expression of MDSCs (Fig. 3c), they increased Arg-1, gp91^phox^, and TGF-β1 expression in PBMC (Fig. 3d). These results demonstrated that UC-MSCs improved the suppressive activity of MDSCs in NOD mice with sialadenitis.

### hUC-MSCs inhibited the differentiation and enhanced the suppressive activity of MDSCs

**Fig. 4:**
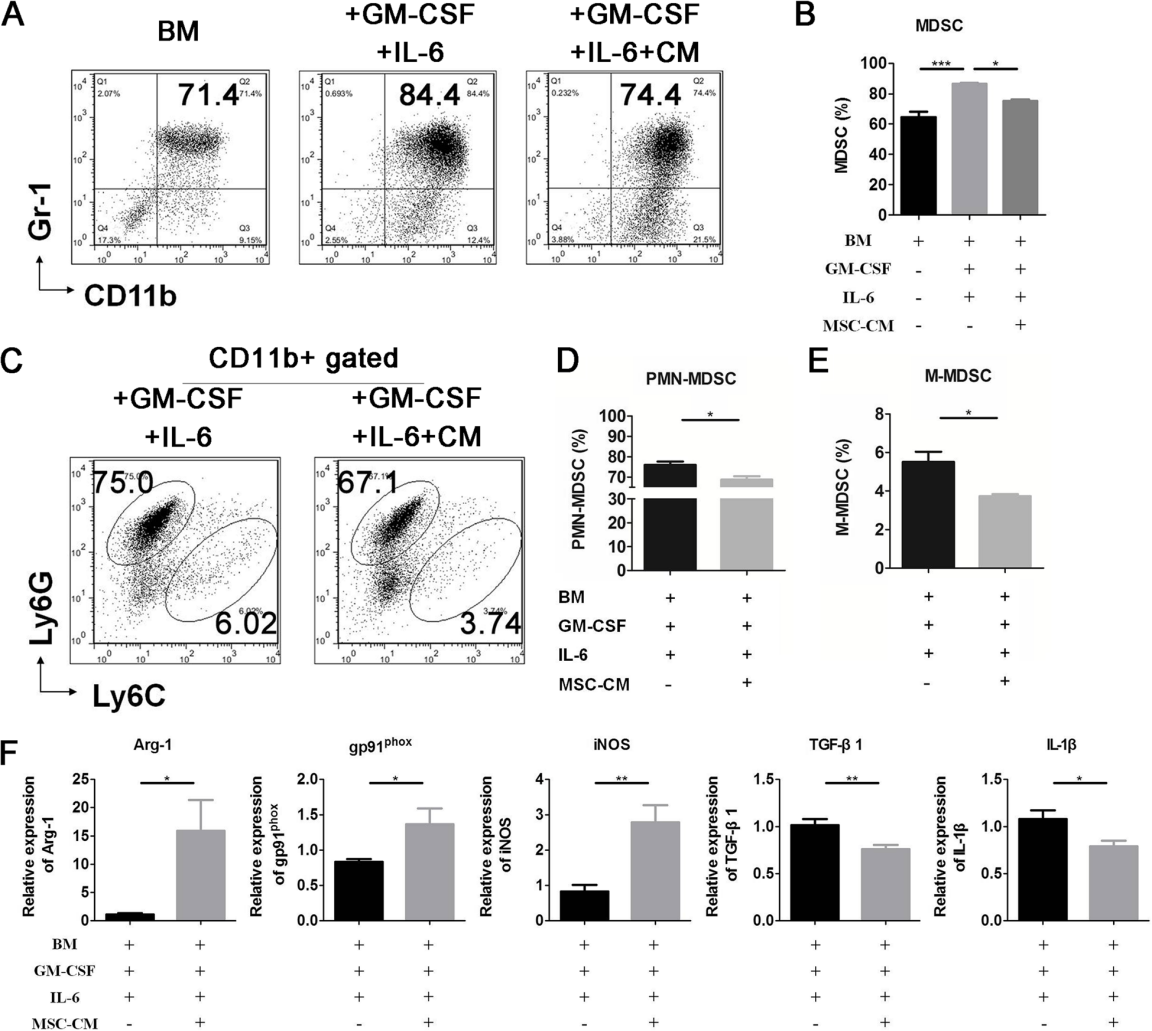
UC-MSCs inhibited the differentiation and enhanced the suppressive activity of MDSCs and the subsets in NOD mice. Bone marrow cells were cultured in RPMI-1640 with 40 ng/ml IL-6 and GM-CSF and with or without UC-MSC-conditioned medium (MSC-CM) for 4 days. **a**, **c** Representative flow cytometry results and percentage of **b** MDSCs, **d** PMN-MDSCs, and **e** M-MDSCs. **f** qPCR results of MDSC suppressive activity genes Arg-1, gp91phox, iNOS, TGF-β1, and IL-1β. Error bars indicate SEM. *p < 0.05, **p < 0.01, ***p < 0.001, *n* = 4

To confirm that hUC-MSCs can suppress MDSC expansion and promote their suppressive ability, we induced bone marrow cells to MDSCs with or without MSC-CM in vitro. The results revealed that MSC-CM inhibited MDSCs (Fig. 4a, b) and the subsets PMN- and M-MDSC (Fig. 4c–e) differentiation significantly. In addition, MSC-CM increased Arg-1, gp91^phox^, and iNOS and decreased TGF-β1 and IL-1β expression in induced BM MDSCs significantly (Fig. 4f). These results indicated that MSCs inhibited MDSC differentiation and promoted MDSC suppressive ability on immune cells by secreting soluble factors.

### hUC-MSCs inhibited the differentiation of PMN-MDSCs and M-MDSCs via COX2/PGE2 and IFN-β, respectively

**Fig. 5:**
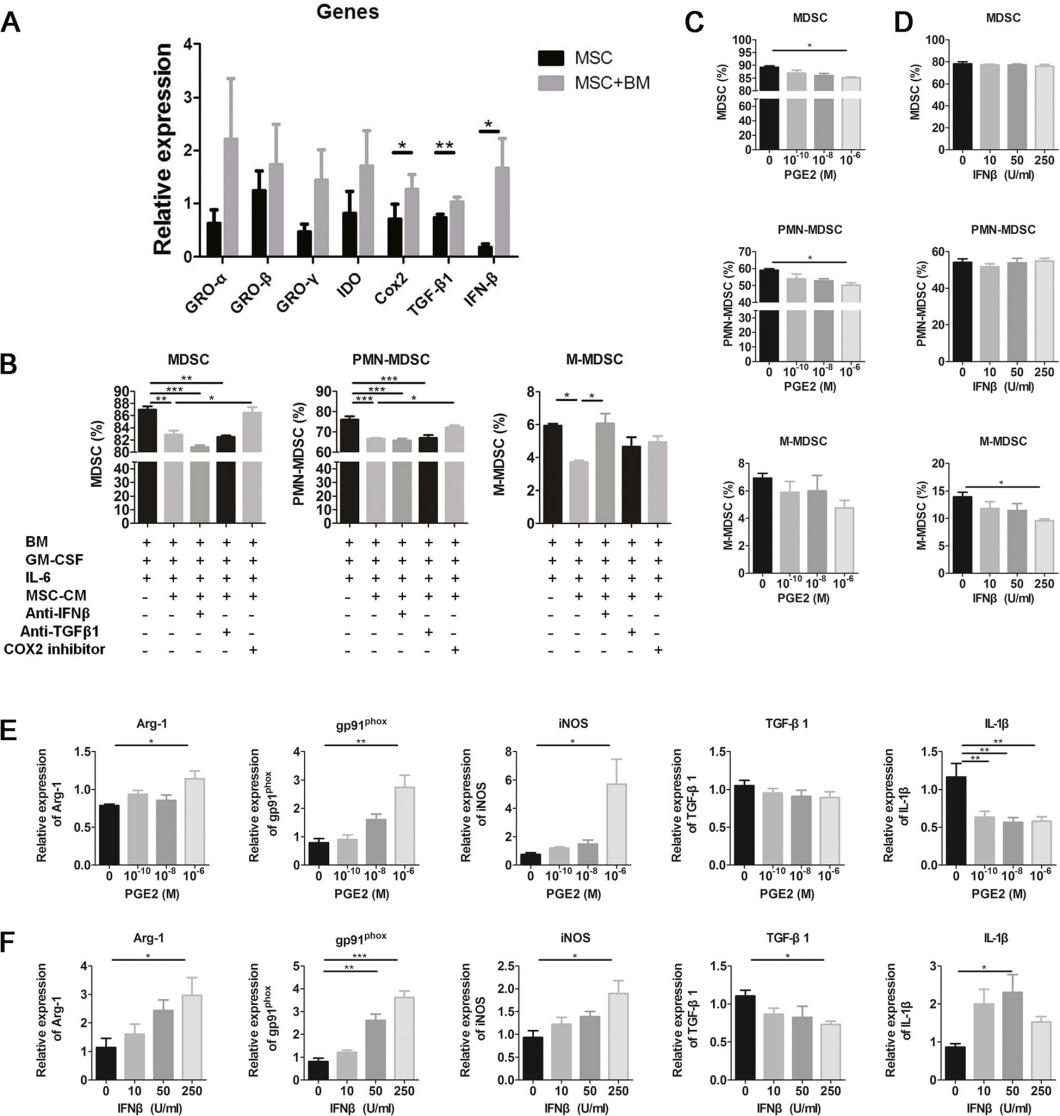
UC-MSCs inhibited the differentiation and enhanced the suppressive activity of PMN-MDSCs and M-MDSCs by COX2/PGE2 and IFN-β, respectively. UC-MSCs co-cultured with bone marrow cells in RPMI-1640 with 40 ng/ml IL-6 and GM-CSF for 4 days by Transwell assays. **a** qPCR results of MDSC differentiation-related genes in UC-MSCs. **b** Bone marrow cells were cultured in MSC-CM with COX2 inhibitor or anti-TGF-β1 or anti-IFN-β antibodies for 4 days. And the percentage of MDSCs, PMN-MDSCs, and M-MDSCs was detected by flow cytometry. **c**, **d** Bone marrow cells were cultured with PGE2 or IFN-β for 4 days. The percentage of MDSCs, PMN-MDSCs, and M-MDSCs was detected by flow cytometry. **e**, **f** MDSC suppressive activity-related genes Arg-1, gp91phox, iNOS, TGF-β1, and IL-1β were detected by qPCR. Error bars indicate SEM. Error bars indicate SEM. *p < 0.05, **p < 0.01, ***p < 0.001, *n* = 4

Since growth-regulated oncogenes (GRO)-α/β/γ, indoleamine 2,3-dioxygenase (IDO), COX2 (the key enzyme for PGE2 production), TGF-β1, and IFN-β have been shown to regulate MDSC differentiation [17, 27, 31], we detected the expression of these molecules in hUC-MSCs co-cultured with or without bone marrow cells under MDSC differentiation conditions. Results show that the co-cultured hUC-MSCs expressed higher levels of COX2, TGF-β1, and IFN-β (Fig. 5a). To determine whether these molecules have suppressive effects on MDSC differentiation, we induced bone marrow cells to differentiate into MDSCs with MSC-CM in the presence of COX2 inhibitor NS-398, anti-TGF-β1, or anti-IFN-β antibodies. Results showed that the COX2 inhibitor could reverse the inhibition effects of MSC-CM on MDSC and PMN-MDSC differentiation and anti-IFN-β antibodies could reverse the inhibition effects of MSC-CM on M-MDSC differentiation (Fig. 5b). Next, we assessed the effects of PGE2 and IFN-β on MDSC differentiation and suppressive ability. We found that PGE2 could inhibit the differentiation of MDSCs and PMN-MDSCs (Fig. 5c) and IFN-β could inhibit the differentiation of M-MDSCs significantly (Fig. 5d). In addition, PGE2 increased the expression of Arg-1, gp91^phox^, and iNOS and decreased the expression of IL-1β in induced BM MDSCs (Fig. 5e). Moreover, IFN-β increased the expression of Arg-1, gp91^phox^, and iNOS and decreased the expression of TGF-β1 and IL-1β in induced BM MDSCs significantly (Fig. 5f). These results demonstrated that UC-MSCs inhibited the MDSC, PMN-MDSC, and M-MDSC differentiation and improved their suppressive ability via COX2/PGE2 and IFN-β, respectively.

## Discussion

**Fig. 6:**
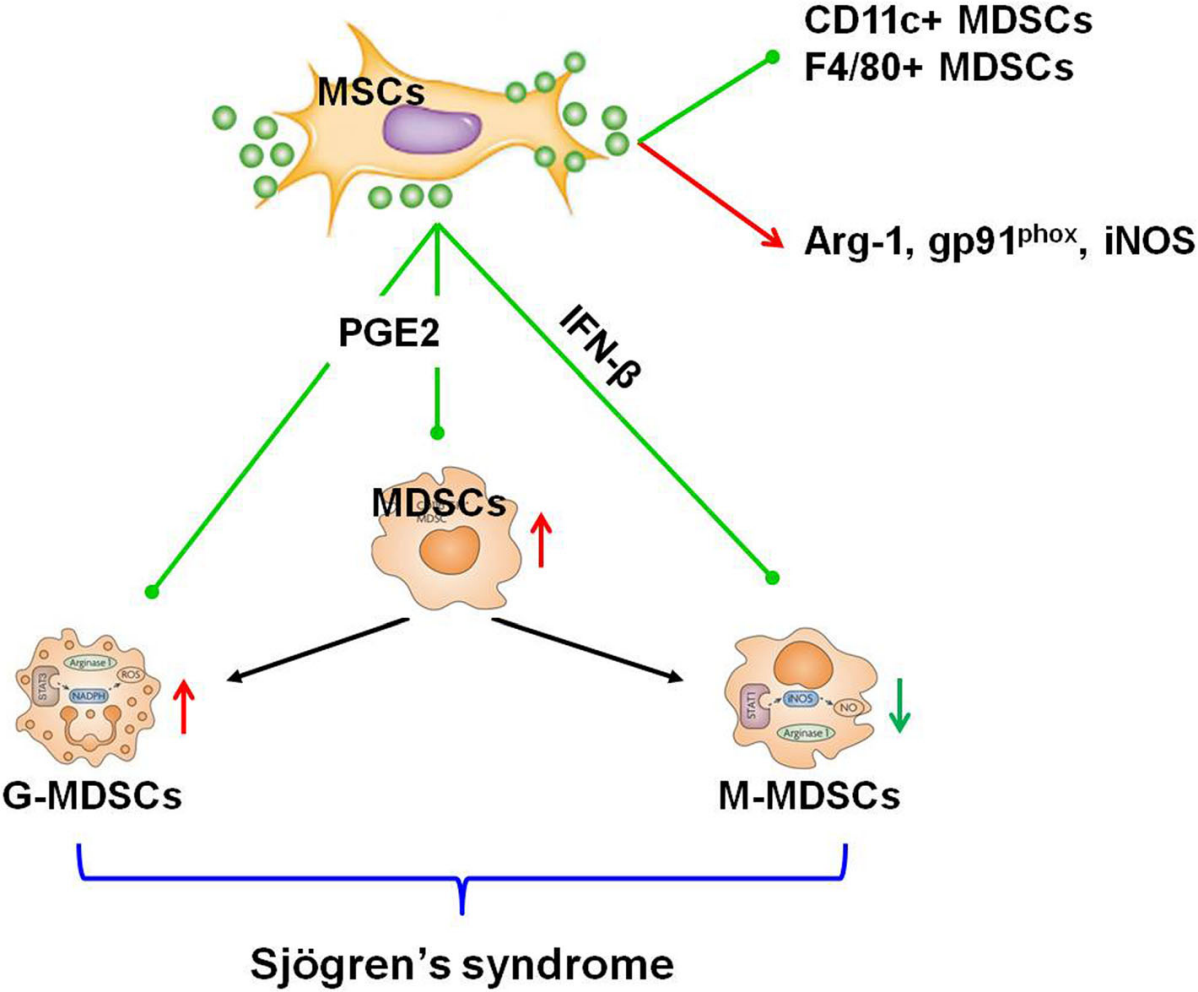
Scheme of MSCs inhibited the differentiation of MDSCs in SS. MDSCs and PMN-MDSCs increased and M-MDSCs decreased significantly in NOD mice with sialadenitis. MSCs ameliorated SS-like syndrome by suppressing the differentiation of MDSCs, PMN-MDSCs, and M-MDSCs via COX2/PGE2 and IFN-β. MSCs rectified the imbalance of PMN-MDSCs and M-MDSCs. And MSCs promoted the suppressive ability by improving the immature or undifferentiated phenotype and increased the Arg-1, gp91phox, and iNOS expression in MDSCs of NOD mice

In the present study, we found that MDSCs accumulated abnormally in NOD mice with sialadenitis. UC-MSCs ameliorated SS-like symptoms by decreasing MDSCs, PMN-MDSCs, and M-MDSCs and promoting their suppressive ability in vivo and in vitro. In addition, hUC-MSCs inhibited PMN-MDSC and M-MDSC differentiation and improved their suppressive ability via COX2/PGE2 and IFN-β, respectively (Fig. 6).

Initially, MDSCs were thought to reflect and affect tumor progression and metastasis, and they could promote immune evasion due to their suppressive activity [32]. Although, a previous study reported that MDSCs showed impaired expansion and function in NZB/W F1 lupus-prone mice [33] and they could control type 1 diabetes by inducing auto-reactive T cells anergy in a diabetic murine model [34], which indicated a protective role of MDSCs. Studies also showed that increased MDSCs were associated with both pathogenetic stress and aging [16]. In recent years, a lot of researchers have found that MDSCs markedly expanded and contributed to progressive disease development in autoimmune disorders, which indicated their pro-inflammatory nature [20–22]. In our previous study, we found that elevated levels of MDSCs exacerbated Sjögren’s syndrome by inhibiting Th2 cell response [23]. These evidences indicated that MDSCs might display different functions in cancer and autoimmune diseases. The appropriate therapeutic strategies aimed at transplantation or elimination of MDSCs under different physiological and pathological conditions should be considered.

MDSCs are heterogeneous cells and identified as two major subpopulations, PMN-MDSCs and M-MDSCs which may play different functions and suppress T cell responses via different mechanisms [19]. Studies reported that M-MDSCs showed more efficient suppressive ability on T cell activation [29]. In our study, the percentages of MDSCs and PMN-MDSCs increased, while M-MDSCs decreased significantly in NOD mice with sialadenitis. UC-MSCs reduced the percentages of MDSCs, PMN-MDSCs, and M-MDSCs in BM and PBMC and the ratio of PMN-MDSCs/M-MDSCs in NOD mice, which implied that hUC-MSCs ameliorated SS-like symptoms by increasing the relative percentage of M-MDSCs and improving their suppressive ability.

Studies have demonstrated that MSCs have extensive immunomodulatory properties on the activation and function of various cells from innate and adaptive immune systems by direct cell-cell contact or secreting soluble molecules [10, 35]. According to studies, MSCs express MHC I and II molecules and can be immune evasive but not immune privileged under inflammatory environments [36]. MSCs may display anti-inflammatory effects in mice, since most transfused MSCs were rapidly phagocytosed by lung-resident macrophages, which induced macrophages to produce IL-10 to regulate immune responses [37]. The live or dead MSC infusion could evoke similar suppressive responses in host mouse lungs [38]. In other words, the suppressive effect of MSCs was independent of their viability.

Regulatory T (Treg) cells are crucial for the maintenance of immunological self-tolerance and homeostasis and play key roles in the prevention of autoimmunity. However, the role of Treg cells in SS remains controversial. Studies reported that the absolute number of circulating CD4+Treg cells in SS patients is significantly lower than that in healthy patients [39]. The female NOD mice develop sialadenitis due to a defect in salivary gland-protective Treg cells [40]. Our previous study also showed that the Treg cells decreased in patients and mice with sialadenitis [41]. However, Alunno and colleagues reported that conventional CD4+CD25highTreg cells decreased, whereas CD4+CD25 lowGITR+Treg cells increased in SS patients [42]. These results suggested that Treg cells play a certain protective role in SS.

hUC-MSCs, an alternative source of MSCs, exhibit a high degree of self-renewal capacity and multi-differentiation potential. Studies have proved the lack of HLA class II antigens and T cell co-stimulatory molecules and express cytokines that may modulate immune function [43]. Both hUC-MSCs and their culture supernatant could inhibit the proliferation of activated human PBMCs and mouse splenocytes [44]. The hUC-MSC immunosuppressive properties have been tested for their therapeutic potential in preclinical animal models since the mid-2000s [11]. Overwhelming evidence showed that hUC-MSCs suppressed immune responses and alleviated various autoimmune diseases in both mouse models and patients [45–48]. These studies suggest that human hUC-MSCs can be a highly safe and effective cell source, which will be tolerated in allogeneic transplantation and have promising clinical application prospects. We have added this section in discussion. In this study, transfused human hUC-MSCs ameliorated experimental sialadenitis by decreasing MDSCs and promoting their suppressive function in NOD mice. MSC-CM inhibited MDSC, PMN-MDSC, and M-MDSC differentiation and improved their suppressive ability via COX2/PGE2 and IFN-β, respectively. However, whether MSC-CM can display similar suppressive function as hUC-MSCs in vivo warrants further study.

A previous study demonstrated that MSCs could protect against diseases through recruiting MDSCs into inflammation sites to suppress CD4^+^ cell proliferation, induce CD4^+^ cell apoptosis [49], promote MDSC expansion [50], tune the differentiation of Gr-1^High^CD11b+ toward Gr-1^Low^CD11b+ cells [51], or tune monocyte-derived dendritic cells to developed toward MDSC phenotype [27]. In our study, MDSCs in BM and PBMC accumulated excessively and play a pathogenetic role in NOD mice. UC-MSCs ameliorated SS by reducing BM and PBMC MDSCs and improving their suppressive ability in NOD mice. The different mechanisms of MSCs in regulating MDSCs may due to the different physiological and pathological conditions, which affected MSC and MDSC functions.

## Conclusions

In conclusion, these data demonstrated that the increased MDSCs may be the crucial pathogenesis of SS, and UC-MSCs ameliorated SS through inhibiting MDSC differentiation and improving their suppressive ability. These findings provided a new view of the mechanisms of MSC transplantation in SS.

## Supplementary information

**Additional file 1: Table S1.** Mouse gene primer sets. **Table S2.** Human gene primer sets.

## Data Availability

The data and materials that support the findings of this study are available from the corresponding authors upon reasonable request.

## Abbreviations

ARG-1: Arginase 1
BM: Bone marrow
COX2: Cyclooxygenase 2
IDO: Indoleamine 2,3-dioxygenase
IFN: Interferon
iNOS: Inducible nitric oxide synthase
GRO: Growth-regulated oncogenes
MDSCs: Myeloid-derived suppressor cells
M-MDSCs: Monocytic MDSCs
MSCs: Mesenchymal stem cells
hUC-MSCs: Human umbilical cord-derived MSCs
MSC-CM: MSC-conditioned medium
NOD: Non-obese diabetic
PBMCs: Peripheral blood mononuclear cells
PGE2: Prostaglandin E2
PMN-MDSCs: Polymorphonuclear MDSCs
ROS: Reactive oxygen
SG: Submandibular glands
SS: Sjögren’s syndrome
TGF: Transforming growth factor
UC-MSCs: Umbilical cord-derived MSCs
