# Supplementary material for: Mesenchymal stem cells inhibited the differentiation of MDSCs via COX2/PGE2 in experimental sialadenitis
Source: Stem Cell Res Ther. 2020 Jul 29;11:325. doi: 10.1186/s13287-020-01837-x (PMC7391592; doi:10.1186/s13287-020-01837-x)
Supplement: Supplementary file 1 — Additional file 1: Table S1. Mouse gene primer sets. Table S2. Human gene primer sets. [file 13287_2020_1837_MOESM1_ESM.doc]

**Mesenchymal Stem Cells Inhibited the Differentiation of MDSCs via COX2/PGE2 in Experimental Sialadenitis**

Jingjing Qi^1, 2^, Xiaojun Tang^1^, Wenchao Li^1^, Weiwei Chen^1^, Genhong Yao ^1^, *, Lingyun Sun^1^, *

^1^ Department of Rheumatology and Immunology, The Affiliated Drum Tower Hospital of Nanjing University Medical School, Nanjing, China.

^2^ Department of Immunology, College of Basic Medical Science, Dalian Medical University, Liaoning, China.

**^*^ Corresponding authors:** Lingyun Sun, MD, PhD, Department of Rheumatology and Immunology, The Affiliated Drum Tower Hospital of Nanjing University Medical School, Nanjing, 210008 China, Email: [lingyunsun@nju.edu.cn](mailto:lingyunsun@nju.edu.cn);

Genhong Yao, E-mail: [yaogenhong@nju.edu.cn](../yaogenhong@nju.edu.cn), Department of Rheumatology and Immunology, Nanjing Drum Tower Hospital，The Affiliated Hospital of Nanjing University Medical School, Nanjing 210093, PR China.

**Conflict of interest:** none

**Financial disclosures/funding sources:** This work was supported by the National Natural Science Foundation of China (NSFC) (grant no. 81770061, 81970062 and 81571583 to Genhong Yao).

[**Supplementary**](javascript:;) [**Materials**](javascript:;)

**Table S1.** Mouse gene primer sets

| Genes | Primer sets |
| --- | --- |
| *Gapdh* | Forward: 5’-GGAGCGAGACCCCACTAA-3’  Reverse: 5’-ACATACTCAGCACCGGCCTC-3’ |
| *gp91^phox^* | Forward: 5’-TCACATCCTCTACCAAAACC-3’  Reverse: 5’-CCTTTATTTTTCCCCATTCT-3’ |
| *arg-1* | Forward: 5’-CTCCAAGCCAAAGTCCTTAGAG-3’  Reverse: 5’-GGAGCTGTCATTAGGGACATCA-3’ |
| *tgf-β1* | Forward: 5’-CCACCTGCAAGACCATCGAC-3’  Reverse: 5’-CTGGCGAGCCTTAGTTTGGAC-3’ |
| *inos* | Forward: 5’-CCTCACCTACTTCC-3’  Reverse: 5’-TGAGGGCTGACACAAGG-3’ |
| *Il-1β* | Forward: 5’-GAAATGCCACCTTTTGACAGTG-3’  Reverse: 5’-TGGATGCTCTCATCAGGACAG-3’ |

**Table S2.** Human gene primer sets

| Genes | Primer sets |
| --- | --- |
| *Gapdh* | Forward: 5’-CGAGATCCCTCCAAAATCAA-3’  Reverse: 5’-TTCACACCCATGACGAACAT-3’ |
| *gro-α* | Forward: 5’-ACTCAAGAATGGGCGGAAAG-3’  Reverse: 5’-TGGCATGTTGCAGGCTCCT-3’ |
| *gro*-β | Forward: 5’-AGCTCTCCTCCTCGCACA-3’  Reverse: 5’-CTTCAGGAACAGCCACCAA-3’ |
| *gro*-γ | Forward: 5’-AGCGTCCGTGGTCACTGAA-3’  Reverse: 5’-AGGTGAATTCCCTGCAGTGTCT-3’ |
| *ido* | Forward: 5’-CGCCTTGCACGTCTAGTTCTG-3’  Reverse: 5’-TGACCTTTGCCCCACACAT-3’ |
| *cox2* | Forward: 5’-CGGTGAAACTCTGGCTAGACAG-3’  Reverse: 5’-GCAAACCGTAGATGCTCAGGGA-3’ |
| *tgf-β1* | Forward: 5’-GCAGAAGTTGGCATGGTAG-3’  Reverse: 5’-CCCTGGACACCAACTATTGC-3’ |
| *ifn-β* | Forward: 5’-AAACTCATGAGCAGTCTGCA-3’  Reverse: 5’-AGGAGATCTTCAGTTTCGGAGG-3’ |
